# Surface Topography, Bacterial Carrying Capacity, and the Prospect of Microbiome Manipulation in the Sea Anemone Coral Model Aiptasia

**DOI:** 10.3389/fmicb.2021.637834

**Published:** 2021-04-08

**Authors:** Rúben M. Costa, Anny Cárdenas, Céline Loussert-Fonta, Gaëlle Toullec, Anders Meibom, Christian R. Voolstra

**Affiliations:** ^1^Division of Biological and Environmental Science and Engineering, Red Sea Research Center, King Abdullah University of Science and Technology, Thuwal, Saudi Arabia; ^2^Department of Biology, University of Konstanz, Konstanz, Germany; ^3^Laboratory for Biological Geochemistry, School of Architecture, Civil and Environmental Engineering, École Polytechnique Fédérale de Lausanne, Lausanne, Switzerland; ^4^Center for Advanced Surface Analysis, Institute of Earth Sciences, University of Lausanne, Lausanne, Switzerland

**Keywords:** coral model system, *Exaiptasia diaphana*, metaorganism, holobiont, microbiome, symbiosis, gnotobiotic, axenic

## Abstract

Aiptasia is an emerging model organism to study cnidarian symbioses due to its taxonomic relatedness to other anthozoans such as stony corals and similarities of its microalgal and bacterial partners, complementing the existing *Hydra* (Hydrozoa) and *Nematostella* (Anthozoa) model systems. Despite the availability of studies characterizing the microbiomes of several natural Aiptasia populations and laboratory strains, knowledge on basic information, such as surface topography, bacterial carrying capacity, or the prospect of microbiome manipulation is lacking. Here we address these knowledge gaps. Our results show that the surface topographies of the model hydrozoan *Hydra* and anthozoans differ substantially, whereas the ultrastructural surface architecture of Aiptasia and stony corals is highly similar. Further, we determined a bacterial carrying capacity of ∼10^4^ and ∼10^5^ bacteria (i.e., colony forming units, CFUs) per polyp for aposymbiotic and symbiotic Aiptasia anemones, respectively, suggesting that the symbiotic status changes bacterial association/density. Microbiome transplants from *Acropora humilis* and *Porites* sp. to gnotobiotic Aiptasia showed that only a few foreign bacterial taxa were effective colonizers. Our results shed light on the putative difficulties of transplanting microbiomes between cnidarians in a manner that consistently changes microbial host association at large. At the same time, our study provides an avenue to identify bacterial taxa that exhibit broad ability to colonize different hosts as a starting point for cross-species microbiome manipulation. Our work is relevant in the context of microbial therapy (probiotics) and microbiome manipulation in corals and answers to the need of having cnidarian model systems to test the function of bacteria and their effect on holobiont biology. Taken together, we provide important foundation data to extend Aiptasia as a coral model for bacterial functional studies.

## Introduction

Corals constitute the foundation species of reef ecosystems that provide a habitat for about a third of all marine life (Fisher et al., 2015), but anthropogenic-driven climate change is now one of the main drivers of coral reef loss (Hughes et al., 2017b, 2018a,b): about half of the Great Barrier Reef corals were lost over the last 30 years (Dietzel et al., 2020) and a 70–99% coral reef decline is projected even under a 1.5–2°C warming, one of the more benign climate change scenarios (Allen et al., 2018). Therefore, it is important to find solutions that can improve coral resilience and mitigate the negative effects of ongoing ocean warming, ocean acidification, and ocean deoxygenation (Allemand and Osborn, 2019; Hughes et al., 2020). Importantly, corals are cnidarian holobionts that consist of the coral animal host, intracellular microalgal symbionts (Symbiodiniaceae), and associated bacteria among many other organisms that all contribute to the stress tolerance and resilience of this metaorganism (Rohwer et al., 2002; Rosenberg et al., 2007; Bang et al., 2018; LaJeunesse et al., 2018; Pogoreutz et al., 2020; Voolstra and Ziegler, 2020). Besides the broad importance of Symbiodiniaceae, which reside within the coral cells and cover almost the entire energy needs of the coral host through provision of photosynthates (Muscatine, 1990; Trench, 1993), bacteria presumably play important roles in metabolism, immunity, and environmental adaptation of the coral host (Ziegler et al., 2017; Ziegler et al., 2019; Robbins et al., 2019; Voolstra and Ziegler, 2020). However, functional studies that detail the specific contributions of specific microbial taxa are still rare, partially due to methodological limitations (Cooke et al., 2019; Robbins et al., 2019). One suggested avenue to elucidate host-bacteria interactions in cnidarians is the use of model organisms, in particular using gnotobiotic (i.e., bearing few remaining known bacteria) or axenic (i.e., bacteria-/germ-free) host systems that allow controlled exposure or provision of cultured bacterial isolates (Jaspers et al., 2019).

Among Cnidarians, the hydrozoan *Hydra* is one of the few models where a germ-free (axenic) closed life cycle is available, which allows the detailed study of associated bacteria and the functions they contribute to the metaorganism, demonstrating the power of such systems to elucidate host-bacterial interactions (Fraune and Bosch, 2007; Fraune et al., 2009; Fraune et al., 2014; Franzenburg et al., 2013b; Augustin et al., 2017; Wein et al., 2018). Similarly, the anthozoan *Nematostella vectensis* is becoming established as a cnidarian model to study host-bacterial interactions (Har et al., 2015; Domin et al., 2018). However, both systems lack the ability to engage in symbioses with microalgal symbionts of the family Symbiodiniaceae (LaJeunesse et al., 2018). Therefore, they may not comprise an ideal model for corals, since association with Symbiodiniaceae affects bacterial assemblage (Ainsworth et al., 2015; Röthig et al., 2016a; Lawson et al., 2018; Maire et al., 2021). To this end, the sea anemone Aiptasia is gaining increasing traction as a coral model due to harboring the same or similar Symbiodiniaceae as scleractinian corals, its simplicity of culturing and clonal propagation, and the fact that Aiptasia anemones can be kept indefinitely in symbiotic and aposymbiotic states (i.e., with and without their microalgal partners) (Weis et al., 2008; Voolstra, 2013), allowing to study the mechanistic underpinnings of the cnidarian-dinoflagellate symbiosis in detail (Baumgarten et al., 2015; Biquand et al., 2017; Cziesielski et al., 2018; Rädecker et al., 2018; Gegner et al., 2019; Simona et al., 2019). Of note, the name Aiptasia refers to the colloquial model system name, given that different researchers across the world use different strains, and likely species (Weis et al., 2008; Baumgarten et al., 2015; Oakley et al., 2015; Biquand et al., 2017; Dungan et al., 2020). The current proposed species name *is Exaiptasia diaphana* (Dungan et al., 2020), previously *Exaiptasia pallida* (Grajales and Rodríguez, 2014). Following the notion of Aiptasia as a model to study the coral-algal symbiosis (Baumgarten et al., 2015), its suitability as a model to study coral-bacterial interactions was suggested (Röthig et al., 2016a). Studies that describe bacterial association of several wild-type and lab-cultured strains could show that (i) microbial assemblages of lab-cultured Aiptasia are comparable, (ii) bacterial associations are somewhat “plastic” pending environmental differences and association with or without algal symbionts, and (iii) an overall similarity in the taxonomic composition of microbiomes of corals and Aiptasia (Röthig et al., 2016a; Brown et al., 2017; Herrera et al., 2017; Dungan et al., 2020). However, a detailed examination of the surface ectoderm topography, bacterial carrying capacity, and the prospect of microbiome manipulation (e.g., in the form of microbiome transplants) to highlight similarities to corals and demonstrate the efficacy of the Aiptasia system as a tool to study bacterial interactions was missing.

In the current work, our aim was to address these knowledge gaps and provide a foundation for Aiptasia to be used as a model for the study of coral-bacterial interactions. To do this, we characterized tissue surface topographies of Aiptasia and the *Hydra* and *Nematostella* cnidarian model systems and subsequently compared them to three scleractinian corals, in the context of the surface ectoderm as a bacterial habitat. In addition, we determined the bacterial carrying capacity in symbiotic and aposymbiotic Aiptasia anemones as a frame of comparison to reef-building corals and to provide a scale of reference for sequencing-based bacterial community studies. Last, using bacteria-depleted sea anemones, we conducted microbiome transplant experiments using coral microbiomes to assess the suitability of this method as a tool to study function of coral-associated bacteria and as a means to assess the ability of changing microbial host association to aid environmental adaptation of coral holobionts.

## Materials and Methods

### Animal Rearing

Aposymbiotic and symbiotic Aiptasia of the clonal strain CC7 were generated and reared as described previously (Baumgarten et al., 2015) with some modifications. Aposymbiotic animals were generated through repeated 4 h cold−shocks at 4°C and treatment with 50 μM of the photosynthetic inhibitor diuron (Sigma−Aldrich, United States). Aposymbiotic animals were maintained in the dark for >3 years and are routinely inspected by fluorescence microscopy (Leica DMI3000 B) to confirm the absence of Symbiodiniaceae. Symbiotic anemones were generated by infecting aposymbiotic animals with strain SSB01 (*Breviolum minutum*, former Clade B) (Xiang et al., 2013). Of note, the algal symbiont strain SSB01 is not the native symbiont of Aiptasia CC7 but has been previously used as a stable and characterized Aiptasia host-algal symbiont combination (Röthig et al., 2016a; Wolfowicz et al., 2016; Simona et al., 2019). To obtain symbiotic CC7-SSB01 Aiptasia, individual anemones were exposed to 10^5^ Symbiodiniaceae cells/ml for 24 h, fed with *Artemia* nauplii after 48 h, and seawater was exchanged thereafter. All Aiptasia anemones were kept in autoclaved natural seawater and reared in 1 L tanks at a 12 h:12 h light:dark cycle at 20–40 μmol photons m^2^ s^1^ of photosynthetically active radiation at 25°C in an I-36LLVL incubator (Percival Scientific Inc., United States). *Nematostella vectensis* polyps were reared in half liter tanks in half strength artificial seawater at room temperature. Polyps of *Hydra magnipapillata* strain 105 were reared in half liter tanks in commercial spring water at room temperature. *Nematostella* and *Hydra* animals were fed *Artemia* nauplii twice a week.

### Ectoderm Surface Analysis Using Electron Microscopy

We compared ectoderm surface topography of different cnidarian classes, namely Anthozoa (corals, Aiptasia, *Nematostella vectensis*) with Hydrozoa (*Hydra magnipapillata*), using Scanning Electron Microscopy (SEM). Coral colony fragments of *Stylophora pistillata*, *Acropora humilis*, and *Porites* sp. were collected from the central Red Sea at 6 m depth at the Al Fahal forereef (22°15.100′N, 38°57.386′E) by SCUBA and maintained in an open water aquaria system until use (CMOR, KAUST), whereas Aiptasia, *N. vectensis*, and *H. magnipapillata* animals were available from cultured lab strains. Three fragments (in the case of corals) or three polyps (in the case of Aiptasia, *Nematostella*, *Hydra*) were transferred to 24-well plates for processing. Coral fragments and model system cnidarians were left to expand in ∼2% magnesium chloride in artificial seawater (ASW) or fresh water for *Hydra*, or half strength ASW for *Nematostella* for 15 min, respectively. After that, specimens were fixed in ∼2.5% glutaraldehyde in 0.1 M cacodylate buffer at 4°C overnight. Samples were then washed in 0.1 M cacodylate buffer (pH 7.2–7.4) thrice for 15 min each and post fixed in 1% osmium tetroxide solution in 0.1 M cacodylate buffer for 1 h in the dark. Samples were further washed thrice in ddH2O for 15 min each and proceeded for dehydration in an EtOH gradient: 30, 50, 70, 90, and 100%, 15 min each step, with two final incubations in 100% EtOH, also for 15 min. The drying process was initiated by transferring the samples to a 1:2 solution of hexamethyldisilazane (HMDS):100% EtOH (v/v) for 20 min, then to a fresh solution of 2:1 HMDS:100% EtOH for 20 min, 100% HMDS for 20 min, and a final incubation in 100% HMDS. Samples were left to dry loosely covered in the chemical hood for the HMDS to slowly evaporate overnight. Each fragment was then mounted on an SEM specimen mount head pin and sputter coated with 4 nm of platinum/palladium or Iridium. All samples were imaged using a Teneo Volume Scope electron microscope (FEI, United States) operating at 1–3 kV.

Complementary to the SEM analyses, we imaged Aiptasia specimens by Transmission Electron Microscopy (TEM). Briefly, Aiptasia polyps were fixed in 2.5% glutaraldehyde in 0.1 M cacodylate buffer (pH 7.4) for 12 h at 4°C and post fixed with 1% osmium tetroxide in 0.1 M cacodylate buffer for 1 h in the dark at 4°C. After three washes in ddH2O, the sample blocks were dehydrated through EtOH and acetone, infiltrated with a mixture of epoxy resin (Electron Microscopy Sciences, United States) and acetone, followed by a final embedding in pure resin. A Leica EM UC6 Ultramicrotome (Leica Microsystems, Germany) was used to cut 150 nm thin sections from the resin block. Finally, thin sections were collected on a 200 mesh copper grid, stained with 1% uranyl acetate (Electron Microscopy Sciences, United States) and Reynold’s lead citrate (Electron Microscopy Sciences, United States). Images were acquired using a Titan CT transmission electron microscope (Thermo Fisher Scientific, United States) operating at 300 kV.

To investigate the presence of bacteria inside the mucus layer using TEM, we modified the standard resin embedding protocol by introducing an agarose embedding step with subsequent cryosectioning of the specimens. Aiptasia anemones were prepared as follows: Individual polyps were chemically fixed in phosphate buffer (0.1 M pH 7.4) with 9% sucrose, containing 4% formaldehyde and 2.5% glutaraldehyde. Fixation was done at room temperature for 2 h after which samples were embedded in 4% aqueous agarose to ease the manipulation. Then, polyps were cut into ∼4 mm^3^ pieces and post stained in 2% osmium aqueous solution for 1 h in the dark. After rinsing in water, samples were dehydrated in a series of ethanol concentrations, ranging from 10 to 100%, then infiltrated with EPON resin (Electron Microscopy Sciences, United States), before polymerization at 60°C for 48 h. Thin sections of 80 nm were cut and mounted onto a Formvar film-coated, carbon-stabilized 100 mesh copper finder grid (Electron Microscopy Sciences, United States). Sections were post stained with UranyLess (Electron Microscopy Sciences, United States) and lead citrate (Electron Microscopy Sciences, United States) before being imaged with a Tecnai-12 transmission electron microscope (Thermo Fisher Scientific, United States) operating at 100 kV with a FEI eagle camera (Thermo Fisher Scientific, United States) using TIA software (Thermo Fisher Scientific, United States). Contrast, brightness, and sharpness of acquired images were adjusted using Adobe Photoshop.

### Generation of Gnotobiotic Aiptasia

Bacteria-depleted Aiptasia polyps were generated using a previously developed protocol consisting of a depletion priming step, followed by an antibiotic treatment, and subsequent recovery from the antibiotic cocktail (Costa et al., 2019). For the depletion priming step, polyps were transferred to 500 ml plastic tanks, reared in 0.22 μm filtered ASW (same light and temperature regime as described above) and fed with sterile decapsulated *Artemia* nauplii for 4 weeks. For the antibiotic treatment, anemones were transferred to petri dishes and washed repeatedly with ASW individually and incubated in antibiotic solution (50 μg/ml of Carbenicillin, Chloramphenicol, Rifampicin, and Nalidixic acid in ASW) for 15 min. Polyps were then transferred to 24-well tissue culture plates (Corning Costar, United States), one polyp per well, under sterile conditions, and incubated with antibiotic solution for 7 days, with daily media exchange, at a 12 h:12 h light/dark cycle in an incubator (20–40 μmol photons m^–2^ s^–1^ of photosynthetically active radiation) at 25°C. After 7 days, anemones were given 24 h for recovery in ASW before microbiome inoculation (see below). Effective bacterial depletion using this protocol was validated using culture-dependent and -independent techniques (Costa et al., 2019): bacterial depletion of treated Aiptasia polyps was confirmed by absence of colony forming units (CFUs) after plating anemone lysates on Marine Agar and subsequent incubation for at least 5 days. In addition, DNA extracted using the DNeasy Blood & Tissue Kit (Qiagen, Germany) from treated Aiptasia anemone lysates were used for PCR amplification of the 16S rRNA gene (95°C for 15 min, followed by 30 cycles of 30 s at 95°C, 90 s at 55°C, and 90 s at 72°C, followed by a final extension for 10 min at 72°C using the 16S universal primer pair 27F-1492R) and subsequent confirmation of absence of a PCR product by means of electrophoresis on an agarose gel.

### Microbial Carrying Capacity

We determined microbial carrying capacity of apo- and symbiotic control Aiptasia polyps (i.e., untreated) as well as of anemones after antibiotic treatment and microbiome transplants. To do this, single polyps from all experimental conditions were placed in 1.5 ml tubes under sterile conditions and 300 μl of ASW were added before the polyps were lysed using a motorized pestle and mortar. Animal lysates and ASW (negative control) were diluted 10-, 100-, and 1000-fold, and 50 μl were plated on Marine Agar (Difco Marine Agar 2216, BD Biosciences, United States) and incubated at 25°C for 24–48 h or 5 days in the case of antibiotic treated anemones, with subsequent counting of bacterial colonies. For statistical analysis, colony counts were log-transformed, normality was tested using the Shapiro–Wilk test, and a one-way ANOVA was conducted. In case of statistical significance, a Dunnett *post hoc* test was conducted. An unpaired *t*-test was used to assess for significant differences between aposymbiotic and symbiotic control conditions. In order to account for putative polyp size differences, CFU counts per polyp were normalized to the host total protein for each polyp, determined using a Micro BCA protein assay kit (Pierce, United States), and the same statistical analysis as above was conducted.

### Microbiome Inocula

Fragments of *Acropora humilis* and *Porites sp.* were collected from the nearshore reef Tahala (22°15.7812′N, 39°3.099′E) (central Red Sea, Saudi Arabia) and processed on the same day. Fragments from three colonies per coral species were collected and combined prior to inoculation (see below). Each coral fragment was placed in a sterile Ziploc bag with 10 ml of ASW, and coral tissue was air blasted off the skeleton using a sterile 1 ml barrier tip inserted to a rubber hose connected to a bench air-pressure outlet. The slurry obtained from fragments from a given coral species were combined and transferred to a 50 ml polypropylene tube and the volume was adjusted to 50 ml with ASW to reduce viscosity. The slurry was homogenized for 30 s using an Ultra Turrax T18 homogenizer (IKA, Germany) and split in 25 ml preparations per tube. Control inocula were prepared using between 10 and 15 apo- and symbiotic Aiptasia polyps, respectively, in 30 ml of ASW, homogenized as described above, and homogenized a second time with a MicroDisTec homogenizer 125 (Thermo Fisher Scientific, United States) to ensure complete maceration. The final volume of the lysate was adjusted to 50 ml with ASW and split in 25 ml preparations.

Microbiome inocula were further processed in a biosafety cabinet, using sterile work practices. Lysates were centrifuged using a swing-bucket rotor at 500 *g* for 5 min to pellet zooxanthellae. The supernatant was collected and an aliquot was taken for visual inspection on an inverted epifluorescence microscope. All lysates were centrifuged once, except for the *Porites* sp. inoculum, which had one extra centrifugation step to completely remove visible traces of the zooxanthellae. The supernatants were pooled for each inoculum type and centrifuged at 3220 *g* for 30 min to pellet bacteria. The resulting pellet was resuspended in 25 ml of ASW and centrifuged twice. Pellets were resuspended in 15 ml of ASW and a 1 ml aliquot was set aside for quantification of bacteria.

Bacteria were quantified using BacLight Red Bacterial stain (Thermo Fisher Scientific, United States). Aliquots from each inoculum were stained using 1 μM of dye for 10 min and counted using a BD FACSCanto II flow cytometer (BD Biosciences, United States). Gates for bacterial counts were first defined using 1 μm and 2 μm reference beads (Thermo Fisher Scientific, United States) in forward scatter (FSC) vs. side scatter (SSC) and then by using Aiptasia bacterial cultures stained with BacLight Red and acquired in the PerCP-PI channel. Bacterial numbers were calculated after gravimetric calibration of the flow rates and using the positive events acquired using the defined gating strategy. Based on determined counts, inocula were diluted to 5 × 10^5^ bacterial cells/ml and 1 ml was used per polyp. In parallel to the flow cytometry-based bacterial counts, an aliquot of all final inocula was plated on Marine Agar (Difco Marine Agar 2216, BD Biosciences, United States) for CFU counts. Bacterial densities of inocula are provided in Supplementary Table 1.

### Microbiome Transplants

For the microbiome transplants, we assessed 30 apo- and symbiotic anemones each across five experimental conditions using six biological replicates (60 anemones in total). Experimental conditions were as follows: (1) untreated control anemones from rearing tanks (APO and SYM), (2) gnotobiotic anemones after 1 day of recovery from antibiotic treatment (APO_AB and SYM_AB), (3) *Acropora* microbiome inoculum (APO+ACRinoc and SYM+ACRinoc), (4) *Porites* microbiome inoculum (APO+PORinoc and SYM+PORinoc), (5) Aiptasia microbiome inoculum (APO+APOinoc and SYM+SYMinoc). Anemones were kept in 24-well plates, 1 polyp per well, and gnotobiotic anemones were inoculated by adding 1 ml of the respective microbiome inoculum to the well (final volume of 1 ml, 5 × 10^5^ bacterial cells/ml) and subsequent incubation for 3 days. After that, anemones were washed twice with ASW and kept in ASW for 4 additional days before being collected (7 days after microbiome transplantation).

### RNA Isolation, cDNA Synthesis, and 16S rRNA Amplicon Sequencing

RNA-based 16S rRNA amplicon sequencing was conducted on five biological replicates from each experimental condition and symbiotic state of the microbiome transplant experiment (see above), in addition to no template DNA extraction and no template PCR negative controls. For RNA isolation, the Qiagen AllPrep DNA/RNA kit (Qiagen, Germany) was used. Briefly, 600 μl of RLT Plus buffer were added to 150 μl of fresh lysate (or artificial seawater for the negative control) followed by snap freezing of the tubes in liquid nitrogen and storage at −80°C until extraction, following the manufacturer’s instructions. RNA quantity and integrity were determined using Qubit (Thermo Fisher Scientific, United States) and BioAnalyzer (Agilent Technologies, United States), respectively. Total RNA was DNase-treated prior to reverse-transcription using the SuperScript First-Strand Synthesis System (Invitrogen, United States), according to the manufacturer’s instructions. For amplification of 16S rRNA amplicons from cDNA, the primers 784F and 1061R (Andersson et al., 2008; Bayer et al., 2013) with MiSeq 16S adapter sequences were used (forward: 5′-TCGTCGGCAGCGTCAGATGTGTATAAGAGACAGAGGA TTAGATACCCTGGTA-3′; reverse: 5′-GTCTCGTGGGCTCGG A GA TGT GTA TA AG A G A C A G CR R C A C GA G CTGACGAC -3′; Illumina overhang adaptor sequences are underlined). PCR reactions were performed in triplicate using the Qiagen Multiplex PCR kit (Qiagen, Germany) with 2 μl of cDNA and a primer concentration of 0.5 μM in a reaction volume of 20 μL. PCRs were performed as follows: 1 cycle at 95°C for 15 min, 27 cycles each at 95°C for 30 s, 55°C for 90 s, and 72°C for 30 s, followed by a final extension step at 72°C for 10 min. Triplicate PCRs for each sample were pooled and cleaned with Illustra ExoProStar (GE Healthcare, United States). Samples were subsequently indexed (eight PCR cycles) using Nextera XT barcode sequencing adapters (Illumina, United States). Indexed PCR products were normalized using Invitrogen SequalPrep normalization plates (Thermo Fisher Scientific, United States) and pooled in equimolar ratios. Pooled samples were checked for the presence of primer dimers on a BioAnalyzer (Agilent Technologies, United States) before sequencing. The library was sequenced at the KAUST Bioscience Core Lab using 2 × 300 bp at 6 pM with 20% phiX on the Illumina MiSeq (version 3 chemistry) according to the manufacturer’s specifications.

### 16S rRNA Amplicon Analysis

Sequence reads were demultiplexed and adapters and barcodes were removed. Resulting sequences were then processed using mothur v.1.39.5 (Schloss et al., 2009). Briefly, paired-end sequences were assembled using the “make.contigs” command, subsequently trimmed to exclude sequences <200 bp, and rare sequences (appearing once across the entire sequencing dataset) were removed. The remaining sequences were then aligned to the SILVA database (version 132) using “align.seqs,” then pre-clustered allowing a 2 nt difference, and finally chimeric sequences were removed using VSEARCH (Rognes et al., 2016). Taxonomical classification was done using the Greengenes (release gg_13_5_99, May 2013) and SILVA (release 138, December 2016) databases. Eukaryotic, archaeal, mitochondrial, and chloroplast sequences were removed prior to OTU clustering using a 97% similarity cutoff. Putative contaminants were determined based on their abundance in negative controls and removed from all samples. An OTU was considered a contaminant if: [Σ relative abundance OTU_*i*_ in negative controls]/[Σ relative abundance OTU_*i*_ in all samples] > 0.1. Beta diversity was examined via principal coordinate analysis (PCoA) of Bray-Curtis dissimilarity distances of log_10_(x + 1) OTU abundances (Supplementary Data 1) using the *phyloseq* package (McMurdie and Holmes, 2013). A permutational multivariate analysis of variance (PERMANOVA) was carried out using the “adonis” function on dissimilarity Bray-Curtis distances of relative OTU abundances to test for differences between conditions and across symbiotic states. Pairwise PERMANOVA tests were conducted using an R wrapper function for multilevel pairwise comparisons (Martinez Arbizu, 2019). To determine overlap of bacterial taxa across microbiome transplants, we determined OTUs that were present across all samples for a given experimental treatment (i.e., APO, APO+APOinoc, ACR+ACRinoc, APO+PORinoc, SYM, SYM+SYMinoc, SYM+ACRinoc, and SYM+PORinoc) and overlapping taxa were represented using the package *VennDiagram* (Chen and Boutros, 2011).

## Results

### Distinct Ectoderm Surface Topographies Across Cnidarians

**FIGURE 1:**
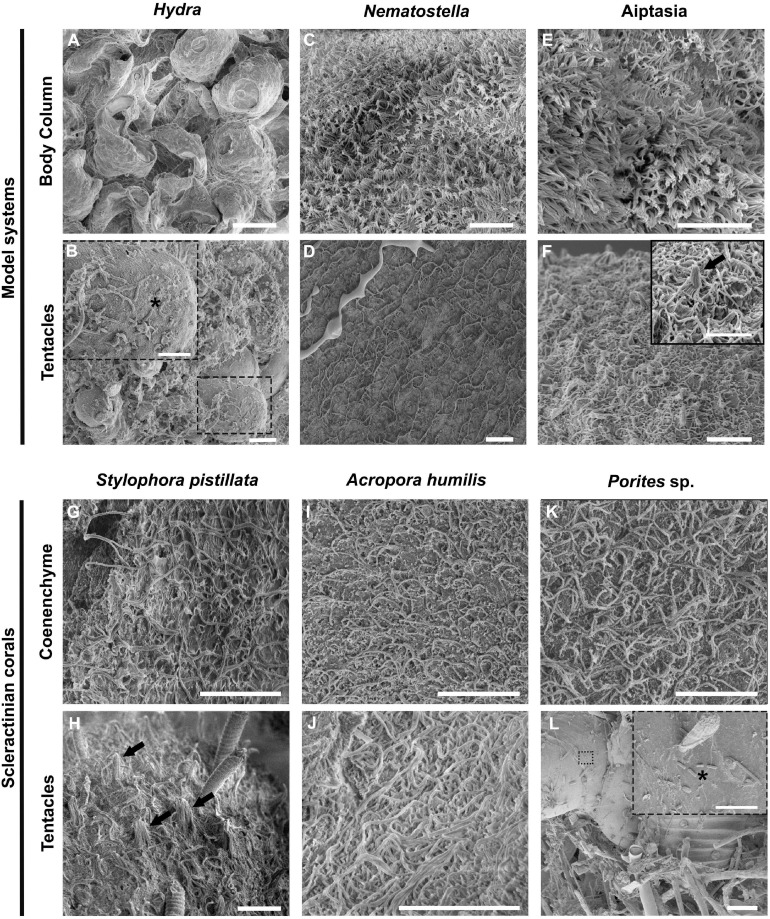
Ultrastructural comparison of the surface topography of *Hydra* and Anthozoans. Representative scanning electron microscopy (SEM) images of three model cnidarians **(A–F)** and three reef building corals **(G–L)** show differences between the hydrozoan *Hydra* and anthozoans, but an overall similar topography across anthozoans. **(A,B)** *Hydra* column and tentacle surface topography. **(C,D)** *Nematostella* column and tentacle surface topography. **(E,F)** Aposymbiotic Aiptasia column and tentacle surface topography. **(G,H)** *Stylophora pistillata* coenenchyme and tentacle. **(I,J)** *Acropora humilis* coenenchyme and tentacle. Noticeable are the discharged cnidocysts in the tentacle. **(K,L)** *Porites* sp. coenenchyme and fouled surface topography. Note the presence of microeukaryotes trapped in the fouled region, to which bacteria seem attached. Dashed line boxes denote regions where bacteria can be seen (black asterisks). Black arrows denote mechanoreceptor bundles. Scale bars: **(A–K)**: 10 μm; **(L)**: 100 μm; boxes in **(B,F,L)**: 5 μm.

**FIGURE 2:**
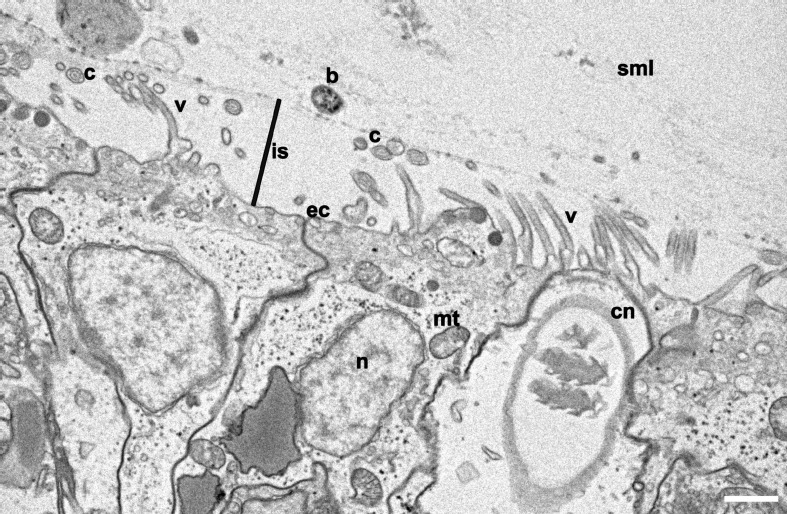
Cross section of the surface ectoderm of Aiptasia. Representative transmission electron microscopy (TEM) micrograph of Aiptasia shows the villi coverage on the epidermis and the interspace between villi and the surface mucus layer (SML), where a bacterium can be identified. b, bacterium; c, cilia; cn, cnidocyte; ec, ectoderm; is, Ectoderm-mucus interspace; mt, mitochondria; n, nucleus; sml, surface mucus layer; v, villus. Scale bar- 1 μm.

We compared surface topographies using scanning electron microscopy (SEM) of three model system cnidarians (*Hydra*, *Nematostella*, and Aiptasia) and three coral species (*Stylophora pistillata*, *Acropora humilis*, *Porites* sp.) as a first proxy to determine microbial association (Figure 1). The ectodermal epithelium of the hydrozoan *Hydra* was composed of a smooth surface with few cilia (i.e., 5–10 μm long hair-like plasma membrane projections, made of microtubules in a 9 + 2 ultrastructure arrangement) (Figure 1A). By comparison, *Hydra* tentacles showed increased ciliation (and a higher density of villi), coinciding with increased mucus and detritus retention, but also exhibited smooth(er) tissue patches scattered across the tentacles, where some bacteria were found to be attached (Figure 1B). The ectodermal epithelium surfaces of column and tentacle of *N. vectensis* and Aiptasia were highly similar (Figures 1C–F). We observed extensive villi (i.e., smaller, numerous plasma membrane projections, lacking microtubules) coverage in the body column of both polyps, with villi protruding from ectodermal cells ranging between 1 and 2 μm in length (Figures 1C,E) and bigger cilia ranging from 4 to 6 μm in the tentacle region (Figures 1D,F). In contrast to *Hydra*, we did not detect any bacteria on the column surface or tentacle regions. This suggests two things: (1) bacteria are rather rare on the surface ectoderm and may be largely constrained to the surface mucus layer in anthozoans, and (2) bacteria may be more abundant on the surface ectoderm in the hydrozoan *Hydra* than in anthozoans. This is corroborated by a complementary TEM analysis of the Aiptasia ectoderm, showing bacteria above the ciliary/villi band, inside the electron dense mucus layer (Figure 2 and Supplementary Figure 1). The preserved surface mucus layer was estimated around 5–15 μm, extending beyond the villi. Of further note, the surface ectoderms of apo- and symbiotic Aiptasia were indiscriminate (Supplementary Figure 2).

We also analyzed the ectodermal epithelium topography of the tentacles and coenenchyme (i.e., coral tissue between the polyps) of the three scleractinian corals *Stylophora pistillata*, *Acropora humilis*, and *Porites* sp. (Figures 1G–I). We observed extensive villi coverage with longer cilia ranging from 4 to 6 μm extending from the epidermis in all corals. Surface topographies were similar to *N. vectensis* and Aiptasia, but distinct from *Hydra*. In the case of *Porites* sp., a thick mucus sheet was visible on top of the coenenchyme, intercalated with fouled areas (Supplementary Figure 3). Further, the surface mucus layer of *Porites* sp. seemed distinct from *S. pistillata* and *A. humilis*, creating a more compact mucus sheet that persisted chemical fixation and several washing steps. Such sheet-like mucus appearances are described for *Porites compressa*, as well as the observation of the presence of fouled regions (Johnston and Rohwer, 2007). Contrary to the coral surface ectoderm that seemed devoid of bacteria, many bacteria were found attached to the smooth epithelial surfaces of other eukaryotes that aggregated in fouled regions of *Porites* sp. (Figure 1L), corroborating the notion that the ciliated surface of anthozoans plays a role in preventing bacterial adhesion.

### Distinct Bacterial Carrying Capacity of Apo- and Symbiotic Aiptasia

**FIGURE 3:**
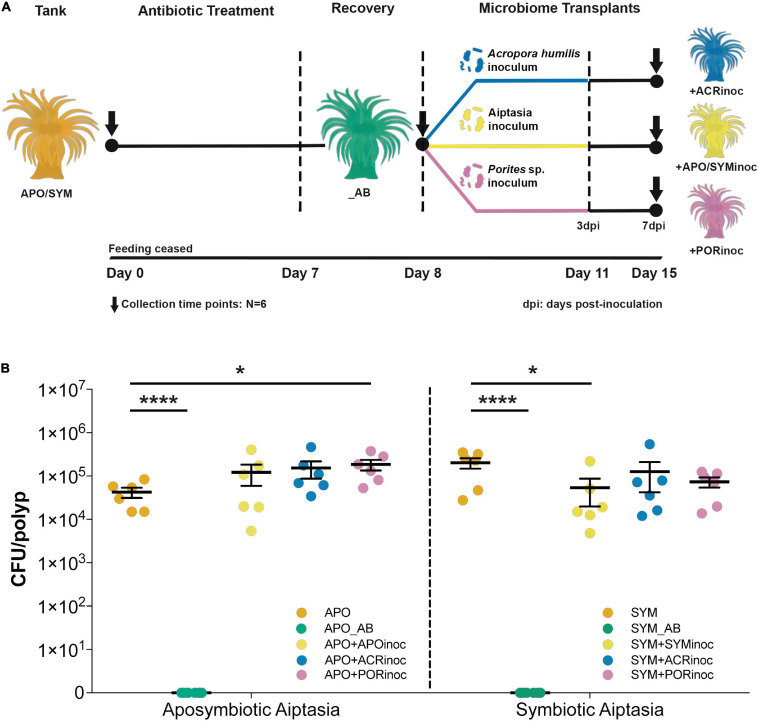
Gnotobiotic Aiptasia, microbiome transplants, and carrying capacity of Aiptasia sea anemones. **(A)** Overview of the microbiome transplant experiment. Anemones underwent antibiotic treatment for 7 days, followed by a 1-day recovery. Animals were then inoculated with preparations of *Acropora humilis*, *Porites* sp., and Aiptasia microbiomes for 3 days, after which inocula were removed (water exchange) and animals were incubated for 4 additional days (total of 7 days). Dotted vertical lines denote a change in the external environment. **(B)** Carrying capacity of Aiptasia sea anemones. CFU counts were determined across all experimental conditions and for apo- **(left)** and symbiotic **(right)** anemones separately. Depicted is the mean and standard error for the respective experimental condition (*P ≤ 0.05; ****P < 0.0001). APO/SYM, apo- and symbiotic control anemones; APO_AB/SYM_AB, 7-day antibiotic-treated anemones with a subsequent 1-day recovery; APO+APOinoc, APO microbiome transplantation after antibiotic treatment; SYM+SYMinoc, SYM microbiome transplantation after antibiotic treatment; APO/SYM+ACRinoc, *Acropora humilis* microbiome transplantation after antibiotic treatment (7 days after inoculation); APO/SYM+PORinoc, *Porites* sp. microbiome transplantation after antibiotic treatment (7 days after inoculation).

To determine the putative carrying capacity of Aiptasia anemones, we obtained CFU counts from apo- and symbiotic control anemones that were subsequently compared to the different experimental conditions, i.e., antibiotic treated and microbiome inoculated animals (Figure 3 and Supplementary Tables 2, 3). The average carrying capacity of aposymbiotic Aiptasia anemones was determined as 4.25 × 10^4^ CFUs/polyp (control animals). When normalized to protein biomass, we obtained 1.59 × 10^5^ CFUs/mg host protein. The carrying capacity of symbiotic anemones was estimated at 2.02 × 10^5^ CFUs/polyp and 1.10 × 10^6^ CFUs/mg host protein, respectively (control animals). Thus, the number of CFUs was higher in symbiotic polyps by about an order of magnitude compared to aposymbiotic polyps (unpaired *t*-test, *P* = 0.025, Supplementary Table 4). We did not obtain CFUs from antibiotic-treated anemones, confirming successful bacterial depletion (Figure 3B). Interestingly, in some cases (re-)infection of gnotobiotic Aiptasia with microbiome inocula of either aposymbiotic Aiptasia or corals increased the carrying capacity of aposymbiotic animals. For instance, aposymbiotic anemones exposed to *Porites* sp. inoculum exhibited a significant increase in CFU counts to an average of 1.85 × 10^5^ CFUs/polyp (Dunnett’s test *P* < 0.05, Figure 3B and Supplementary Tables 2, 4). Conversely, inoculation of symbiotic anemones (SYM+ACRinoc and SYM+PORinoc) did not result in a significant increase in CFU counts (Dunnett’s test *P* > 0.3, Figure 3B and Supplementary Tables 2, 4). Overall, the carrying capacity of Aiptasia polyps with ∼5 mm of oral disk was between 10^4^ and 10^5^ CFUs for apo- and symbiotic anemones, respectively. After microbiome inoculation the carrying capacity was at about 10^5^ CFUs/polyp, irrespective of the symbiotic condition.

### Bacterial Community Composition of Native and Inoculated Aiptasia

**FIGURE 4:**
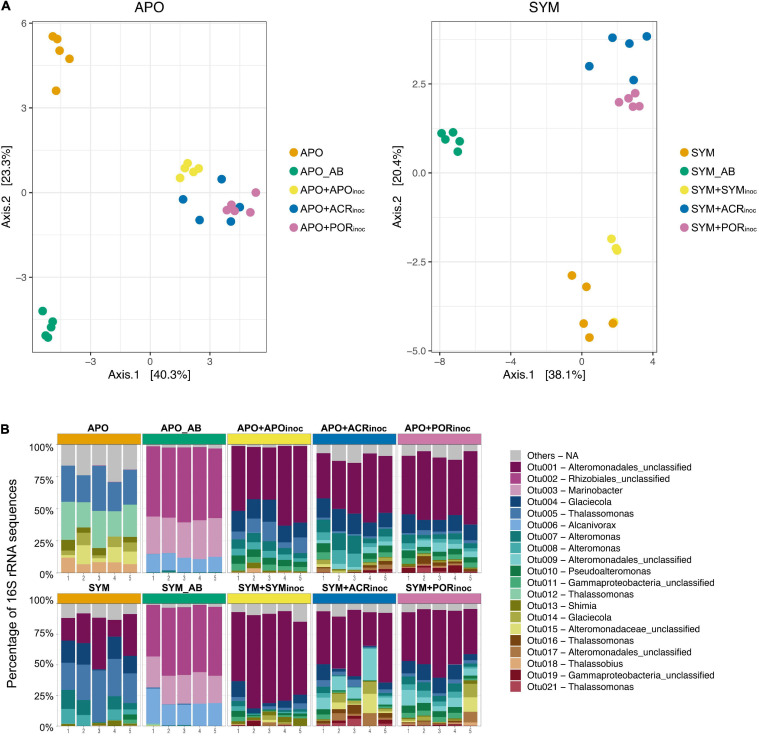
Bacterial community composition and dynamics of Aiptasia polyps 7 days after microbiome transplantation. **(A)** Principal Coordinate Analysis (PCoA) based on Bray-Curtis distances of OTU log_10_(x + 1)-transformed abundances. **(B)** 16S rRNA amplicon-based active bacterial community composition at the OTU level. Depicted are the 20 most abundant (i.e., active) OTUs across all samples (refer to Figure 3A for an overview over time points sampled). Remaining OTUs are grouped as “Others.” APO/SYM, apo- and symbiotic control anemones; APO_AB/SYM_AB, 7-day antibiotic-treated anemones with a subsequent 1-day recovery; APO+APOinoc, APO microbiome inoculation after antibiotic treatment; SYM+SYMinoc, SYM microbiome inoculation after antibiotic treatment; APO/SYM+ACRinoc, *Acropora humilis* microbiome inoculation after antibiotic treatment (7 days after inoculation); APO/SYM+PORinoc, *Porites* sp. microbiome inoculation after antibiotic treatment (7 days after inoculation).

We employed RNA-based 16S amplicon sequencing to assess active bacterial community composition and dynamics (in contrast to the resident community based on DNA-based 16S sequencing) associated with Aiptasia under the various experimental treatments (Figure 4). Bacterial community composition differed significantly between treatments (PERMANOVA, *P* = 0.001) (Supplementary Table 5). As previously reported based on DNA-based 16S marker gene sequencing (Röthig et al., 2016a), bacterial communities of apo- and symbiotic Aiptasia anemones were different (PERMANOVA, *P* = 0.015, Figure 4B). For this reason, we clustered apo- and symbiotic samples separate to resolve differences within apo- and symbiotic groups as a result of the treatments (Figure 4A). For both groups, antibiotic-treated anemones (APO_AB and SYM_AB) were clearly separated from control anemones (APO and SYM) and both were different from gnotobiotic anemones re-inoculated with microbial communities (with the exception of SYM+SYMinoc that closely clustered with SYM anemones) (Figure 4A).

The bacterial assemblage of antibiotic-treated anemones (APO_AB and SYM_AB) was markedly different from the microbiomes of other anemones in that it was considerably less complex. This was highlighted by the notion that >90% of the active community in apo- and symbiotic anemones was comprised of the same three bacterial taxa (OTU0002, OTU0003, and OTU0006) affiliated to the order Rhizobiales and the genera *Marinobacter* and *Alcanivorax* (Figure 4B). Conversely, 75% of the active community of aposymbiotic control anemones was comprised of only six bacterial taxa (OTUs 0005, 0012, 0013, 0014, 0015, 0018, Figure 4B), which were largely absent in the antibiotic treated Aiptasia. Notably, >70% of the bacterial communities of all inoculated aposymbiotic anemones were dominated by the same taxa regardless of the inoculum. The dominant bacterial taxa were representatives of the order Alteromonadales (OTU0001, OTU0004, OTU0007, OTU0008), which were barely detected in aposymbiotic control anemones. For the symbiotic anemones, the active bacterial community of control polyps was dominated by a few bacterial taxa of the order Alteromonadales and the genera *Glaciecola* and *Thalassomonas* (Figure 4B). Similarly, microbiome transplanted symbiotic Aiptasia resembled each other and were dominated by bacteria of the order Alteromonadales (OTU0001, OTU0004, OTU0007, OTU0008, OTU0009), just as the microbiome transplanted aposymbiotic Aiptasia. Thus, despite the differences in microbial community composition of apo- and symbiotic control anemones, microbiome transplanted gnotobiotic apo- and symbiotic anemones look much more alike with regard to their microbiome.

**FIGURE 5:**
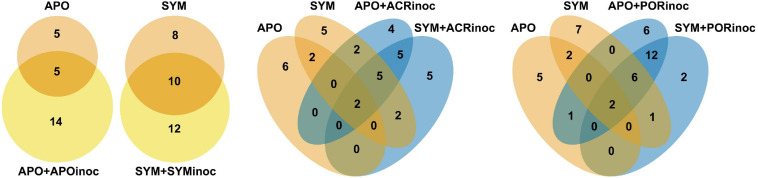
Bacterial taxa of control and microbiome transplanted Aiptasia. Venn diagrams representing the number of active OTUs ubiquitously present in all control anemones (APO/SYM), shared between APO/SYM anemones and transplanted anemones (APO+APOinoc, APO+ACRinoc, APO+PORinoc and SYM+SYMinoc, SYM+ACRinoc, SYM+PORinoc), and ubiquitously present in transplanted anemones 7 days after the microbiome transplantation. In the absence of sequenced inocula we chose to assess bacterial taxa that were consistently present in coral inoculated Aiptasia, but not present in control Aiptasia, as an indication for bacteria of coral origin. APO/SYM, apo- and symbiotic control anemones; APO_AB/SYM_AB, 7-day antibiotic-treated anemones with a subsequent 1-day recovery; APO+APOinoc, APO microbiome transplantation after antibiotic treatment; SYM+SYMinoc, SYM microbiome transplantation after antibiotic treatment; APO/SYM+ACRinoc, *Acropora humilis* microbiome transplantation after antibiotic treatment (7 days after inoculation); APO/SYM+PORinoc, *Porites* sp. microbiome transplantation after antibiotic treatment (7 days after inoculation).

To get better insight into the bacterial taxa that were present in Aiptasia after microbiome transplantation, we compared the active bacterial community of control conditions (APO/SYM) and treatment conditions (Figure 5 and Supplementary Table 6). This comparison shows that all inoculated anemones (+APOinoc, +SYMinoc, +ACRinoc, and +PORinoc) harbored bacterial taxa that were already present (active) in control anemones as well as “novel” (i.e., previously undetected) bacteria. This may point to potential carryover from gnotobiotic anemones (not all bacteria were depleted). In the case of coral microbiome inoculations, we observed association with novel bacteria from the coral microbiomes (as determined by the comparison to APO and SYM anemones, Figure 5 and Supplementary Table 6). In particular, two bacteria taxa, of the genus *Thalassomonas* (OTU0016) and an unclassified Alteromonadales (OTU0021), are worthwhile mentioning because they were both found to be abundant (i.e., active) in coral microbiome transplanted Aiptasia (i.e., APO+ACRinoc, APO+PORinoc, SYM+ACRinoc, and SYM+PORinoc), but absent from Aiptasia transplanted with their own microbiomes (i.e., APO+APOinoc and SYM+SYMinoc) (Supplementary Table 6). Both OTUs are putative coral microbiome bacteria that were able to colonize apo- and symbiotic Aiptasia. On the contrary, a putative coral microbiome taxon from the genus *Glaciecola* (OTU0195) was only found associated with symbiotic anemones. Further, we found two Gammaproteobacteria (OTU0067 and OTU0099) to be specifically prevalent in anemones inoculated with *Porites* sp. microbiome preparations.

## Discussion

It is becoming increasingly clear that the specific composition and abundance of certain bacterial species affect host health and fitness (McFall-Ngai et al., 2013). At the same time, recent studies highlight that bacterial associations are not stable over time, but rather, assemble flexibly following fluctuations in the prevailing environment(s) (Roder et al., 2015; Ziegler et al., 2017; Ziegler et al., 2019; Voolstra and Ziegler, 2020). On the one hand, such observations support the notion that microbiome community changes can support host ecological adaption to environmental change (Reshef et al., 2006; Voolstra and Ziegler, 2020). On the other hand, the premise of flexibility gives rise to the idea of probiotics, i.e., inoculation with beneficial bacteria to support metaorganism stress resilience (Peixoto et al., 2017, 2019, 2021). The latter is gaining attention with regards to reef-building corals given the alarming loss of coral reef cover over recent decades (Hughes et al., 2017a, 2020). However, besides the acknowledged importance of coral-associated bacteria (Bang et al., 2018; van Oppen and Blackall, 2019) and the promise of coral probiotics to work in principle (Rosado et al., 2019), we are still missing answers to many of the basic questions surrounding cnidarian microbiome building principles and the underlying mechanistic aspects. Aiptasia is an emerging model system to study cnidarian symbioses, and here we set out to build a foundation for bacterial functional studies using the Aiptasia model by characterizing surface topography, carrying capacity, and assessing the prospect of microbiome transplants.

Our results show pronounced differences in the ultrastructure of the surface ectoderm between *Hydra* and anthozoans with implications for microbial association. SEM analyses of *Hydra*, *N. vectensis*, Aiptasia, and three scleractinian coral species suggest that bacterial epibionts in anthozoans are (more) restricted to the surface mucus layer (SML) in comparison to hydrozoans. Notably, *Hydra* anemones feature a smooth surface ectoderm where the epithelial glycocalyx (i.e., the pericellular matrix made of glycoproteins and glycolipids that cover the plasma membrane of the epithelium) promotes bacterial attachment and lacks extensive ectodermal ciliation that may prevent bacterial adhesion (Kesimer et al., 2013; Fraune et al., 2014; Wein et al., 2018). Other hydrozoans such as *Ectopleura crocea* and *Cladonema* sp. have shown colonizing epibionts attached to the glycocalyx, which ranged from 200 nm to 1 μm in thickness from the hydroid ectoderm to the epibiont, and their surface topography was also shown to be smoother, resembling that of Hydra (di Camillo et al., 2012; Abouna et al., 2015). Although *Hydra* can be considered a derived hydrozoan due to its life history (e.g., lack of a medusoid stage and freshwater habitat), imaging results from the current and other studies (e.g., di Camillo et al., 2012; Abouna et al., 2015) support the general notion that hydrozoans and anthozoans differ in their surface topographies, with anthozoans featuring a ciliated, rough surface, and a more defined ectoderm-SML separation in contrast to hydrozoans that seem to exhibit a smooth(er) less ciliated surface. It has been shown that the SML of corals is highly dynamic, with SML being cyclically shed (Bythell and Wild, 2011). This is thought to prevent pathogen colonization from the surrounding environment (Shnit-Orland and Kushmaro, 2009; Glasl et al., 2016), but might also explain the flexible microbial association of corals commonly found across different environments (Roder et al., 2015; Röthig et al., 2016b, 2017; Ziegler et al., 2017; Voolstra and Ziegler, 2020) and may contribute to the efficacy of coral probiotics (Peixoto et al., 2017, 2021). At a more basal level, the difference in surface topography between hydrozoans and anthozoans argues for the need of having distinct cnidarian models to reflect the implied microbial association differences. For the two anthozoan models, *N. vectensis* and Aiptasia, there was little difference in terms of ectodermal topography and bacterial colonization between the two organisms, both at the column and tentacle level. However, microalgal symbionts in the family Symbiodiniaceae (LaJeunesse et al., 2018) were shown to putatively contribute to the composition of coral mucus through their exudates (Brown and Bythell, 2005; Nelson et al., 2013), which affect bacterial association (Matthews et al., 2020), and Symbiodiniaceae were also shown to harbor themselves specific bacteria (Maire et al., 2021). As such, Aiptasia is a model system not only for the study of coral-algal symbiosis, but also for the study of bacterial associations and for testing the capacity for microbiome manipulation as further discussed below.

We were surprised by the lack of observed bacterial colonization at the surface ectoderm of Aiptasia (anthozoans more generally) using SEM, which was readily apparent in *Hydra* using the same technique. As alluded to above, this might be a consequence of surface topography differences, which ultimately affect bacterial colonization, but also differences in how the preparation affects sample integrity. In the case of *Hydra*, the accessible glycocalyx was preserved during SEM preparation, whereas the mucus of the anthozoans species was lost by employing a classical SEM sample protocol. Indeed, a modified TEM protocol developed to preserve the mucus layer also confirmed the presence of bacteria in the surface mucus layer of Aiptasia polyps, but not in the ectodermal layer or glycocalyx (Supplementary Figure 1). This highlights the need for continuous development and improvements of protocols to visualize bacterial association (Wada et al., 2016). For instance, TEM analyses may be combined with CARD-FISH staining for improved specificity and visualization of Aiptasia- and coral-associated bacteria besides the exploration of other methods (Kesimer et al., 2013).

Complementary to the visual assessment of bacterial abundance, we determined a bacterial carrying capacity of ∼ 10^4^ to 10^5^ bacterial cells per Aiptasia polyp, pending on the symbiotic state. This number is similar to the bacterial density reported for *Hydra* (Wein et al., 2018) and may roughly equate to the ∼10^6^ bacterial cells/cm^2^ coral tissue previously reported (Koren and Rosenberg, 2006; Garren and Azam, 2012). It is interesting to note that our bacterial capacity was an order of magnitude higher for symbiotic (10^5^ bacterial cells/polyp) in comparison to aposymbiotic (10^4^ bacterial cells/polyp) anemones. This difference may arise from the additional niche space provided by the symbiosome, which was previously shown to harbor bacteria (Ainsworth et al., 2015), and through association with Symbiodiniaceae, which harbor their own microbial community (Deines et al., 2020; Maire et al., 2021). Visualization and enumeration of cnidarian-associated bacteria is still relatively rare (Neave et al., 2017; Cooke et al., 2019), in part because of the difficulties associated with fluorescent staining techniques in corals (Wada et al., 2016). As such, we relied on CFU counts, which avoid many of these difficulties but at the cost of media selectivity, as highlighted by the discrepancy between absence of CFUs in marine media and amplicon sequencing-based detection of some bacterial taxa. Literature perusal suggests that the most prevalent bacterial taxa detected in gnotobiotic anemones using sequencing (OTU0002, OTU0003, and OTU0006) cannot grow on our employed media and therefore escape CFU counting (Brooijmans et al., 2009; Yetti et al., 2015; Wang et al., 2016). Nevertheless, the antibiotic-treated anemones were highly bacteria depleted as more than 95% of the sequencing reads represented only three OTUs, which correspond to less than 5% of the active taxa detected in control anemones. As such, we consider animals treated with our gnotobiotic protocol (Costa et al., 2019) highly bacteria depleted. Besides the availability of axenic or gnotobiotic animals, it is desirable to have bacterial isolates with fluorescent reporter plasmids for more accurate estimation, which also allows for tracking location and abundance of said bacteria, as shown in *Hydra* (Wein et al., 2018). On this note, viability-qPCR (Emerson et al., 2017) may comprise a molecular method for enumeration of the density of active bacteria, although we found that it requires significant optimization due to taxon-specific differences with regard to dye permeability. As alluded to above and recently (Röthig et al., 2016a), 75% of the relative microbial abundance is comprised of only a handful of bacterial taxa, making Aiptasia a “non-complex” coral model for microbiome studies with the promise to obtain bacterial isolates for functional testing and manipulation (Röthig et al., 2016a).

Our RNA-based sequencing analysis revealed that the community of active bacteria was less diverse in comparison to the DNA-based microbial community (Röthig et al., 2016a): we identified an average of 38 and 64 active bacterial taxa associated with apo- and symbiotic anemones, while DNA-based analysis of the standing community retrieved 109 and 118 distinct bacteria, respectively. This may seem like a stark difference; however, it is not straight-forward to compare RNA- and DNA-based bacterial communities. Since abundance estimates are relative, “absence” in the RNA-based community analysis merely indicates that we could identify less bacteria (at the current sequencing depth) and that there is a putative difference between the bacterial community that is “present” (DNA) and the one that is “active” (RNA). Nevertheless, we compared the overlap in OTU-assigned taxonomies at the genus level for CC7 from Röthig et al. (2016a) and this study, which showed that 65% (APO) and 84% (SYM) of the identified bacterial genera in the RNA-based analysis were also found in the DNA-based analysis. Notably, such comparisons have inherent biases due to taxonomic redundancies and differences in the taxonomic classification between different OTU datasets.

To begin to explore the prospect of Aiptasia microbiome manipulation as a tool to interrogate bacterial function and test the effect of probiotics on holobiont biology, we conducted a series of inoculations/transplants on gnotobiotic Aiptasia with microbiomes from control anemones and from two coral species. *Acropora humilis* was chosen because its microbiome is highly uneven and dominated by bacteria of the genus *Endozoicomonas*, which could be used for tracking of the microbial transplant, since Aiptasia CC7 seems devoid of *Endozoicomonas* (Röthig et al., 2016a). However, we did not detect *Endozoicomonas* in the microbiome of inoculated anemones (i.e., SYM+ACRinoc, APO+ACRinoc). This suggests that *Endozoicomonas* exhibit high (coral) host specificity, despite their broad and prevalent distribution across marine invertebrates (Neave et al., 2016, 2017; Rossbach et al., 2019). Moreover, *Endozoicomonas* reside within coral tissues (Neave et al., 2016, 2017), which may explain their absence after microbiome transplantation, because mucus-associated bacteria may be easier to transfer than tissue-associated bacteria. Such differences putatively provide important insight regarding the choice of bacteria targeted for microbiome transplants. In contrast to *A. humilis*, *Porites* sp. was chosen as a donor because it is considered an environmentally resilient species with a more even and diverse microbiome (Hadaidi et al., 2017; Robbins et al., 2019). As such, successful microbiome transplantation would allow for subsequent testing of altered stress susceptibility. First off, our results show that all transplanted animals harbor a significantly different active bacterial community when compared to control anemones with the general notion that inoculated Aiptasia resemble each other more than control animals (Figure 4 and Supplementary Table 5). This is not highly surprising given that formation of an established microbial community may take time and likely goes through processes of inter-bacterial communication, host-bacterial communication, and winnowing, all of which presumably affect microbiome composition (Franzenburg et al., 2013a; Bernasconi et al., 2019; Shibl et al., 2020). As such, microbial consortia associated with Aiptasia after microbiome transplantation might represent a mix of specific bacteria administered with the inocula and opportunistic, environmentally present bacteria (e.g., resistant bacteria that survived the antibiotic treatment and were attached to the wells of the rearing plate), in particular copiotrophs. Copiotrophs are known to rapidly (re)populate carbon-rich environments such as the surface mucus layer (Nelson et al., 2013; McDevitt-Irwin et al., 2017; Cárdenas et al., 2018; Hadaidi et al., 2019) and as such may “drive” initial microbial repopulation dynamics. This makes the long(er)-term tracking of microbiome assemblage dynamics after transplantation important. It also suggests that antibiotic treated Aiptasia without any subsequent inoculation should be included in future experimental designs as controls for determining load and type of “residual” bacteria. In addition, future experiments should also include microbiome transplantations of untreated Aiptasia, resembling current coral probiotics procedures (Peixoto et al., 2021). This would provide further insight regarding repopulation dynamics and constraints.

Many of the taxa found in control Aiptasia established themselves again after microbiome inoculation, such as bacteria in the genus *Thalassomonas* (OTU0005, OTU0012), *Glaciecola* (OU0014), *Thalassobius* (OTU0018), or *Alteromonas* (OTU0007) (Supplementary Table 6). Microbiome transplantations with inocula from *A. humilis* and *Porites* sp. were successful to the extent that (at least) some foreign bacterial taxa (some of which were specific to the coral species from which the inoculum was obtained) were present (active) in Aiptasia polyps, as evidenced by their detection 7 days after microbiome transplantation. Notably, unavailability of sequenced inocula and coral native microbiomes within the current study limited the extent to which we could identify bacterial taxa with broad host compatibility, suitable for cross-species microbiome manipulation. Due to this, we considered bacterial taxa that were consistently present in coral-inoculated but not present in control Aiptasia as of putative coral origin. Besides treatment-specific differences, we commonly found bacterial taxa belonging to the Alteromonadaceae, Rhodobacteraceae, and Gammaproteobacteria to be transferred. This is encouraging given that bacteria from these taxonomic affiliations are commonly found in coral microbiomes (Roder et al., 2014; Neave et al., 2017; Ziegler et al., 2017, 2019).

At large, our results suggest that cross-species microbiome manipulation via transplantation is possible (to a certain extent). That is to say, Aiptasia is a suitable system to test the function of (at least some) coral bacteria and their effect on holobiont biology. The obvious next step is to test for altered holobiont phenotypes, e.g., increased or decreased stress resilience, using a standardized framework (Voolstra et al., 2020) after microbial transplantation with subsequent elucidation of the underlying mechanism(s). In addition, to achieve consistent and stable changes of microbial host consortia, an improved understanding of inocula persistence, dispersion, location, bacterial load, and any putative long-term effects is wanted. Aiptasia seems like the model system of choice, given its taxonomic relatedness and physiological similarities with regard to Symbiodiniaceae association, microbiome composition, and surface and tissue complexity.

## Conclusion

Bacteria affect the health and fitness of their hosts. Given the worldwide decline of corals and the reefs they build, a better understanding of the function of bacteria and their potential manipulation is important. To achieve this, the development of coral model systems is imperative. Here we set the foundation for Aiptasia as a model for the study of coral-bacterial interactions. We show that the surface ectoderm topography is highly similar between Aiptasia and corals, in line with overall similarities in the microbiome composition established previously. Building on these prospects, we have developed a protocol for the generation of gnotobiotic Aiptasia and determined the bacterial carrying capacity to perform microbiome manipulation experiments. Our results support the principal suitability of Aiptasia to microbiome manipulation and its putative ability to incorporate foreign bacterial species. Although more work is needed, our study is a first step and provides an avenue to study the function of self and foreign bacteria as well as to explore the mechanisms underlying microbial acquisition, association, and host specificity. Future work should incorporate standardized testing to elucidate the effect of altered microbiomes on holobiont phenotypes as well as the generation of fluorescently labeled bacterial isolates, which will allow for spatial/temporal tracking and enumeration of bacterial associates. The work presented here provides a foundation and we will continue to develop Aiptasia as a coral model for bacterial functional studies.

## Data Availability Statement

Data determined in this study are available under NCBI BioProject PRJNA665377 (https://www.ncbi.nlm.nih.gov/bioproject/665377). Scripts used for data analysis, curation, and plotting are available at https://github.com/ajcardenasb/Aiptasia_microbiome_manipulation. Abundant bacterial OTU reference sequences are available under GenBank Accession numbers MW577132 – MW577168 (https://www.ncbi.nlm.nih.gov/nuccore/?term=MW577132:MW577168[accn]).

## Author Contributions

CV, RC, and AC conceived and designed the experiments, analyzed the data, and wrote the manuscript. RC and AC collected and processed the samples. RC, AC, and CL-F generated the data. RC, AC, CL-F, GT, and CV generated the figures. RC, AC, CL-F, GT, AM, and CV interpreted the data. CL-F, AM, and CV provided tools, reagents, and methods. All authors contributed to the article and approved the submitted version.

## Conflict of Interest

The authors declare that the research was conducted in the absence of any commercial or financial relationships that could be construed as a potential conflict of interest.
