# Supplementary material for: Surface Topography, Bacterial Carrying Capacity, and the Prospect of Microbiome Manipulation in the Sea Anemone Coral Model Aiptasia
Source: Front Microbiol. 2021 Apr 8;12:637834. doi: 10.3389/fmicb.2021.637834 (PMC8060496; doi:10.3389/fmicb.2021.637834)
Supplement: Supplementary file 3 [file Image_1.pdf]

Supplementary Figures

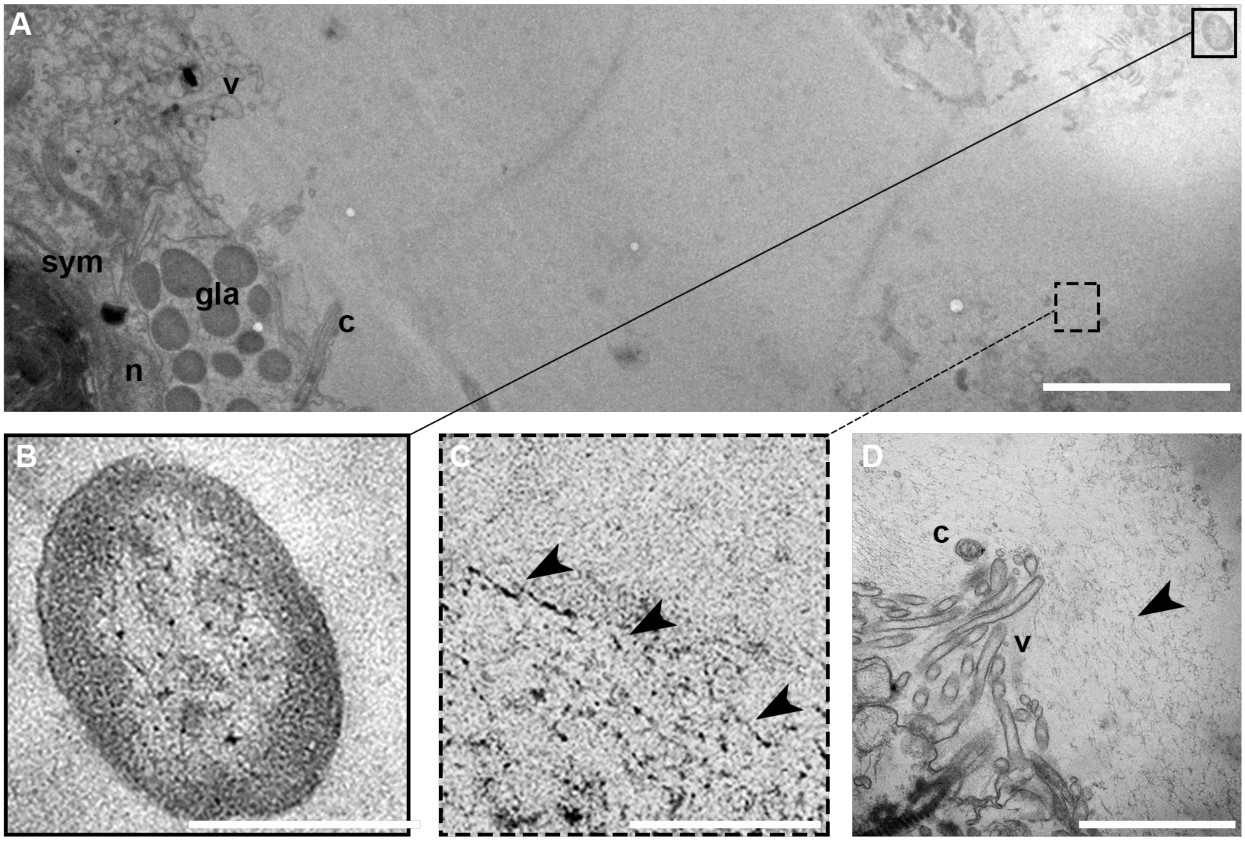

Supplementary Figure S1. Cross-section of the surface ectoderm of Aiptasia employing a mucus-preserving protocol. (A) Representative transmission electron micrograph of the ectodermal epithelium and ectoderm-mucus interspace. (**B**) A bacterium is visible surrounded by preserved mucus. **(C)** Preserved mucus (black arrows) presented as electron dense streaks extending from the ectoderm. **(D)** Surrounding bundles of villi and cilia in the epithelium. c – cilia; gla – granular gland cells; n – nucleus; sym- Symbiodiniaceae; v - villus. Scale bars: 5 µm in **(A)**; 500 nm in **(B)** and **(D)**; 250 nm in **(C)**.

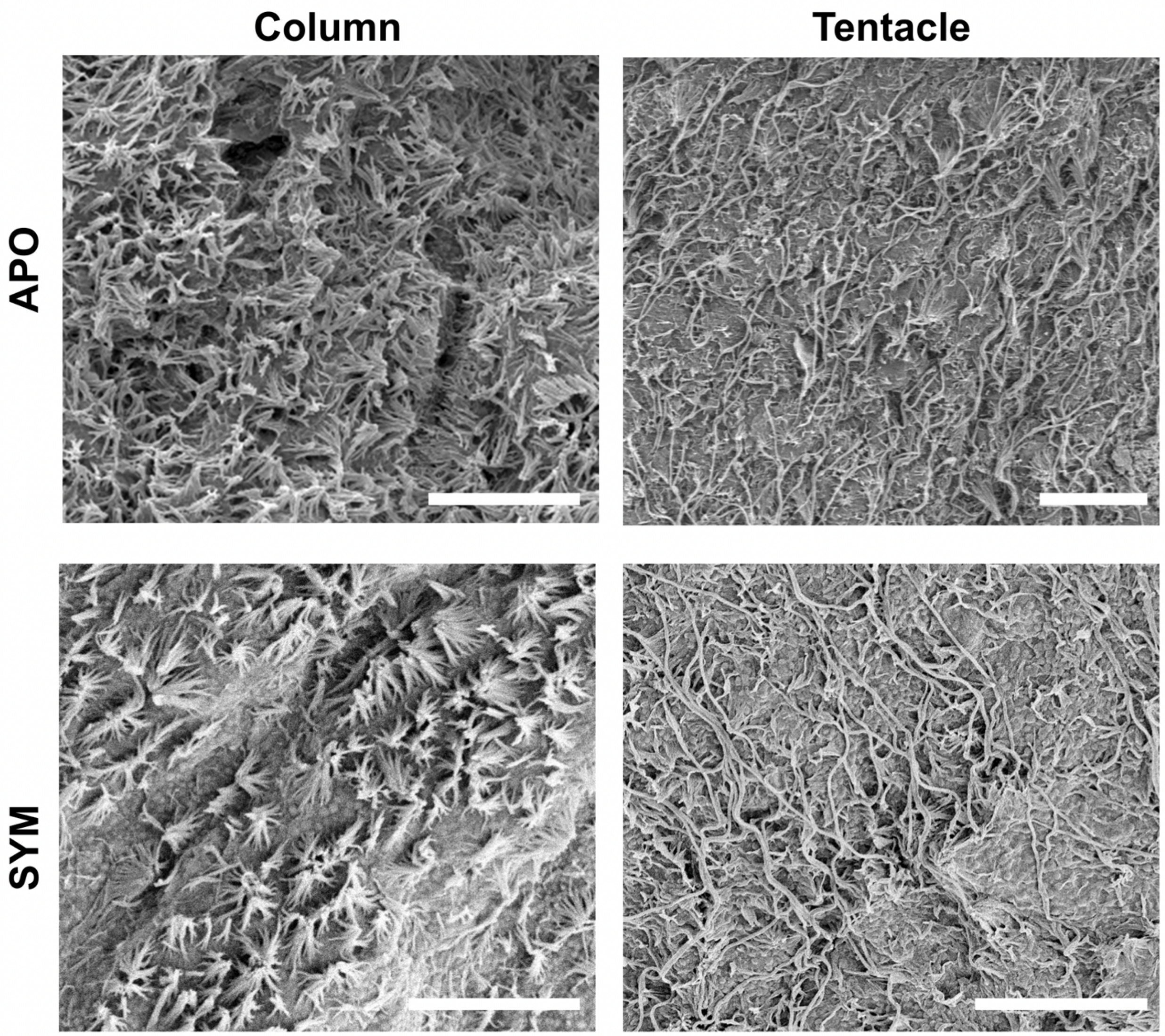

Supplementary Figure S2. Ultrastructural comparison of surface topography of aposymbiotic and symbiotic Aiptasia polyps. Representative scanning electron micrographs of the tentacle and column regions of Aiptasia polyps in different symbiotic states, showcasing similar surface ectodermal ultrastructures. Scale bars: 10 µm.

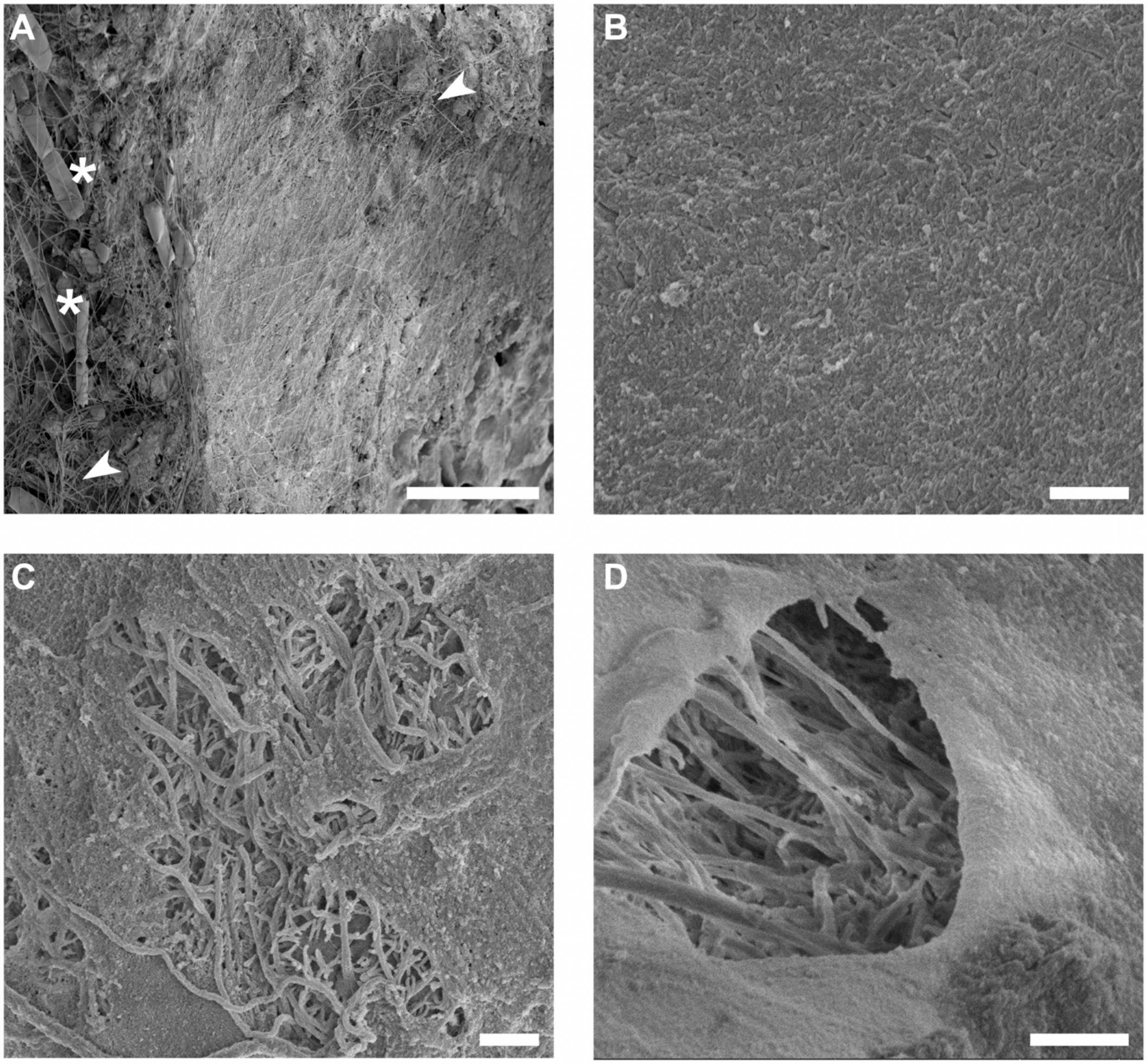

Supplementary Figure S3. Topography of fouled regions and mucus sheets in *Porites* sp. ectodermal surface. **(A)** Representative scanning electron micrograph of fouling present throughout *Porites* sp. surface, where pennate diatoms are visible (white asterisks), as well as filaments belonging to filamentous algae or fungi (white arrows). **(B)** Close-up of a mucus sheet spread across the coenenchyme. **(C)** Ciliated ectoderm of the coenenchyme, seen in areas where the mucus sheet is discontinuous. **(D)** Close-up of the ciliated ectoderm of a tentacle of a polyp, concealed by the thick mucus sheet. Scale bars: 500 µm in **(A)**; 2 µm in **(B)**, **(C)**, and **(D)**.
